# Intracerebroventricular injection of ouabain causes mania-like behavior in mice through D2 receptor activation

**DOI:** 10.1038/s41598-019-52058-z

**Published:** 2019-10-30

**Authors:** Alexander Lopachev, Anna Volnova, Anna Evdokimenko, Denis Abaimov, Yulia Timoshina, Rogneda Kazanskaya, Olga Lopacheva, Alex Deal, Evgeny Budygin, Tatiana Fedorova, Raul Gainetdinov

**Affiliations:** 1 Research Center of Neurology, Volokolamskoye shosse 80, 125367 Moscow, Russia; 2 Institute of Translational Biomedicine, Saint Petersburg State University, Universitetskaya Emb. 7/9, 199034 St Petersburg, Russia; 3 Biological Department, Saint Petersburg State University, Universitetskaya Emb. 7/9, 199034 St Petersburg, Russia; 4 Biological Department, Lomonosov Moscow State University, Leninskiye Gory 1, 119991 Moscow, Russia; 5 International Biotechnological Center, Lomonosov Moscow State University, Leninskiye Gory 1, 119991 Moscow, Russia; 6 Department of Neurobiology and Anatomy, Wake Forest School of Medicine, Medical Center Boulevard, Winston-Salem, 27157 North Carolina USA

**Keywords:** Biochemistry, Kinases, Neurochemistry

## Abstract

Intracerebroventricular (ICV) administration of ouabain, an inhibitor of the Na, K-ATPase, is an approach used to study the physiological functions of the Na, K-ATPase and cardiotonic steroids in the central nervous system, known to cause mania-like hyperactivity in rats. We describe a mouse model of ouabain-induced mania-like behavior. ICV administration of 0.5 µl of 50 µM (25 pmol, 14.6 ng) ouabain into each lateral brain ventricle results in increased locomotor activity, stereotypical behavior, and decreased anxiety level an hour at minimum. Fast-scan cyclic voltammetry showed that administration of 50 µM ouabain causes a drastic drop in dopamine uptake rate, confirmed by elevated concentrations of dopamine metabolites detected in the striatum 1 h after administration. Ouabain administration also caused activation of Akt, deactivation of GSK3β and activation of ERK1/2 in the striatum of ouabain-treated mice. All of the abovementioned effects are attenuated by haloperidol (70 µg/kg intraperitoneally). Observed effects were not associated with neurotoxicity, since no dystrophic neuron changes in brain structures were demonstrated by histological analysis. This newly developed mouse model of ouabain-induced mania-like behavior could provide a perspective tool for studying the interactions between the Na,K-ATPase and the dopaminergic system.

## Introduction

In recent years, there has been an increasing number of reports on physiological and pathological processes in which the Na,K-ATPase plays an important role. Na,K-ATPase is a membrane protein which creates and maintains the electrochemical gradient across the plasma membrane of animal cells. In the central nervous system (CNS), the catalytic α subunit of Na,K-ATPase is represented by three isoforms; α1 is present in all cells of a mammalian body, α2 is expressed only in glial cells and myocytes, and α3 is present only in neurons of adult mammalian organisms^1^. α3 may act as a receptor of endogenous cardiotonic steroids (CTS) in the CNS^1–3^. CTS are a group of steroid-based compounds, most of them act as specific inhibitors of Na,K-ATPase^4^. Endogenous CTS are involved in various physiological and pathological processes, such as blood pressure regulation, cardiotonic effect, natriuretic effect, development of hypertension and preeclampsia, etc.^2,3,5–7^. However, to date, the functions of endogenous CTS in the CNS are poorly studied, though it is well-known that treatment of heart failure with high doses of CTS digoxin may cause mood disturbances^8^. Moreover, a more recent study has shown that Na,K-ATPase activity is compromised in patients with bipolar disorder^9^. There is a lot of data which demonstrates the association of Na,K-ATPase dysfunction with neurological and neuropsychiatric disorders^10,11^. Recent studies have demonstrated that α-synuclein^12^ and β-amyloid^13^ oligomers can inhibit Na,K-ATPase function, which may be one of the mechanisms underlying the relationship between Na,K-ATPase function and neurodegenerative processes. CTS ouabain is known to activate several signaling cascades in neurons, such as MAPK^14,15^, Akt^16^, PKC^17^, etc. Several studies have demonstrated that ouabain can modulate the function of the dopaminergic system by binding to Na,K-ATPase that functionally interacts with dopamine receptors^18^. Microdialysis studies have also shown that 100 μM ouabain injected locally into the rat striatum causes an increase in extracellular striatal concentration of dopamine (DA) and 3,4-dihydroxyphenylacetic acid (DOPAC), and decreases that of glutamate and acetylcholine^19^. It is also known that riluzole, a neuroprotective agent effective in models of parkinsonism and other neurodegenerative diseases, dose-dependently increases the release of dopamine caused by ouabain due to stabilization of the Na^+^ channels in the closed state^20^.

In 1995, а model of ouabain-induced mania-like behavior in rats was developed. In this model intracerebroventricular (ICV) injection of ouabain instantly caused hyperactivity in rats^21^. The ouabain-induced model of mania-like behavior in rats is recognized among the employed pharmacological models of mania^22,23^. Also, ICV ouabain administration has an effect on cognitive flexibility and reversal learning in rats^24^. Administration of the anti-ouabain antibodies attenuates the increase in locomotor activity, which is accompanied by a threefold increase in brain CTS induced by amphetamine. Consequently, malfunctioning of the Na,K-ATPase, as well as endogenous CTS, may be involved in the manifestation of mania and identifies this system as a potential new target for drug development^25^. Mutations in the α3 isoform cause mania-like behavior in mice^26^. Since hyperactivity, stereotypic behavior, and decreased anxiety in rodents are believed to recapitulate certain endophenotypes of psychiatric disorders, such as bipolar disorder and schizophrenia^27–29^, several studies using antimanic and antipsychotic drugs were performed on this model^30,31^. It also provided a tool for studies of the physiological role of Na,K-ATPase and CTS in the CNS. It has been demonstrated that the effects of ouabain in this model occur due to activation of ERK1/2, Akt and tyrosine hydroxylase^16,32^ indicating the involvement of the dopaminergic system in the development of ouabain-induced hyperactivity. Furthermore, hyperactive dopamine transporter knockout (DAT-KO) mice with an increased amount of extracellular dopamine are considered to be a model for psychiatric disorders associated with enhanced dopaminergic transmission^33–35^, which further suggests that ouabain causes hyperactivity by decreasing dopamine reuptake in the striatum. Recently it has been shown that mice become hyperactive 7 days after ICV administration of 0.625 nmol of ouabain^36^, however, to date, there has been no model of mania-like behavior in mice similar to the aforementioned rat model, where animals become hyperactive immediately after ouabain injection. Such a model could provide evidence of Na,K-ATPase interaction with the dopaminergic system on an object different from the rat model, as well as a more rapid and less expensive tool for studying the interaction between the Na,K-ATPase and the dopaminergic system. It would also enable the use of transgenic mice, with mutations in genes directly or indirectly affecting the Na,K-ATPase, in studying the physiological and pathological roles of Na,K-ATPase and CTS in the CNS. In this report, we describe a novel mouse model of acute ouabain-induced mania-like behavior. The contribution of the dopaminergic system in the observed effects was also evaluated.

## Results

### Increased locomotor activity induced by ouabain in mice

After two days of rehabilitation prior to the administration of ouabain or aCSF, experimental animals were placed into the open field for a 1 h habituation to measure the basal locomotor activity.

**Figure 1:**
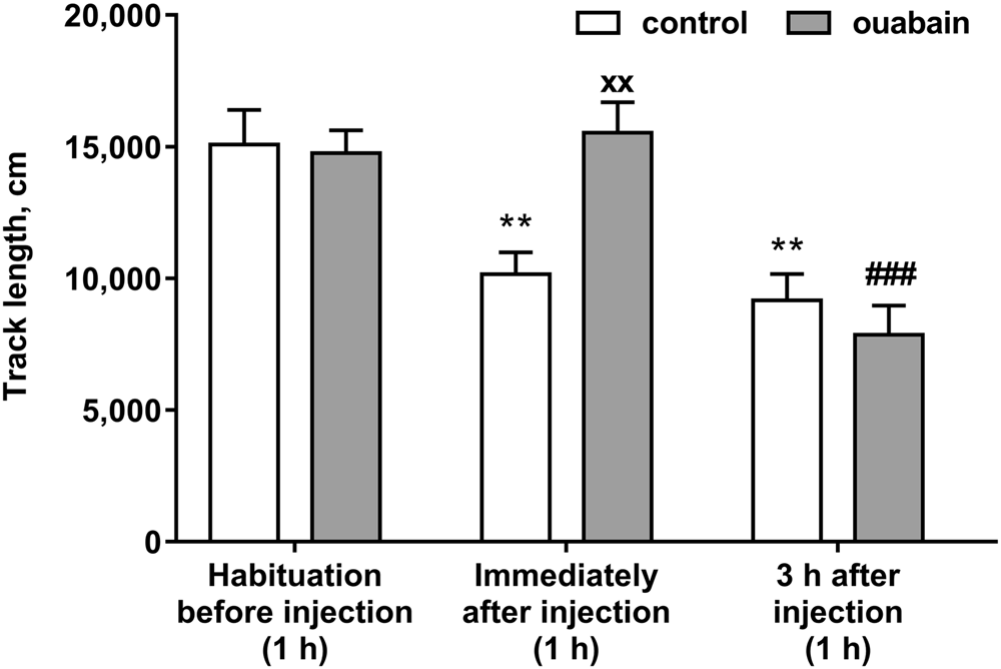
Total distance travelled by mice in the 1 h open field tests. Data are presented as mean ± SEM, N = 10 per group, ^×^p < 0.05, ^××^p < 0.01 compared to control; **p < 0.01, ***p < 0.001 compared to control/habituation; ^#^p < 0.05, ^##^p < 0.01 compared to ouabain/habituation according to two-way ANOVA with Tukey’s multiple comparisons test.

Animals were separated into groups with similar locomotor activity after habituation (p > 0.9999, q = 0.3123) (Fig. 1). To evaluate alterations in the locomotor activity of the animals after aCSF or ouabain injection we performed open field tests for 1 h immediately after the injection, and 3 h after the injection. Administration of 0.5 μl of 50 μM ouabain into each lateral ventricle resulted in a 1.53-fold increase in locomotor activity in the open field test during 1 h immediately after the injection (p = 0.0116, q = 4.989) (Fig. 1). There was no difference in locomotor activity between the control and ouabain-treated mice 3 h after the injection (p = 0.9928, q = 1.127) (Fig. 1). In the control group locomotor activity decreased after habituation on the same day, by 1.49 times immediately after the injection (p = 0.0301, q = 4.68) and by 1.64 times 3 h after the injection (p = 0.0078, q = 5.326). In the ouabain-treated group, locomotor activity was not significantly different in 1 h open field test immediately after the injection compared to habituation (p = 0.9996, q = 0.7264), but decreased by 1,88 times 3 h after the injection (p = 0.0010, q = 6.212).

In all three open field tests, as well as during habituation, locomotor activity subsequently decreased with time both in the control and in ouabain-treated groups, as well as hyperactivity in ouabain-treated group maintains along 1 h after injection compared to control group, but there was no significant changes between groups 3 h after injection and 24 h after injection (see Supplementary Fig. S5).

However, since animal models of mania-like behavior are characterized not only by hyperactivity, but also stereotypic behavior and decreased anxiety, proof of hyperactivity alone is not enough to verify the model. As such, we have conducted additional tests in order to evaluate the degree of stereotypical behavior and decreased anxiety in the ouabain group as compared with the control group.

### Evaluation of the anxiety and stereotypical behavior of animals in the elevated plus maze

**Figure 2:**
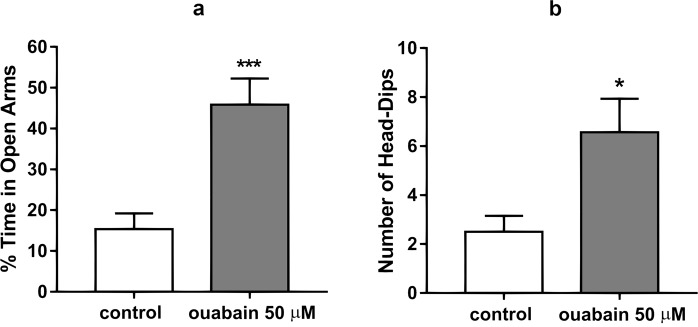
(**a**) Percent of time spent in open arms during the five-minute long elevated plus maze test by the control and ouabain-treated groups. (**b**) Number of head-dips from the open arms of the elevated plus maze for the duration of the five-minute long test for the control and ouabain treated groups. Results are presented as mean ± SEM, N = 8 per group; *p < 0.05, ***p < 0.001 compared to control according to unpaired t-test.

The elevated plus maze test was performed to evaluate anxiety and stereotypical behavior of the animals after the ICV injections. The ouabain-treated group spent more time in the open arms of the maze and less time in the closed arms compared to the control group, thus demonstrating a decreased anxiety level. Under the action of ouabain, the percentage of time spent in the open arms increased from 15.38 ± 3.84% in the control group to 45.86 ± 6.40% (Fig. 2a). Apart from the increase of time spent in the open arms, head-dipping was recorded for both the ouabain-treated and control groups. The number of head-dips from the open arms of the maze in the control group was 2.50 ± 0.66, while in ouabain-treated the number of head dips was 6.57 ± 1.37 (p = 0.0003, t = 4.08) (Fig. 2b). After ouabain administration, animals were prone to falling or jumping off of the open arms of the elevated plus maze, which was not observed in the control group. The demonstrated increase in time spent in the open arms, as well as the increase in head-dips, shows that the ouabain-treated group showed a decrease in anxiety and an increase in stereotypical behavior in comparison with the control group. Having registered an increase in locomotor activity, stereotypical behavior, and decreased anxiety in animals post ouabain injection compared with the control, we had grounds to propose that these effects were realized through the activation of the D2 dopamine receptor.

### Blockade of the D2 dopamine receptor prevents ouabain-induced hyperactivity and stereotyped movements in mice

To determine whether the ouabain-induced increase in locomotor activity, stereotypic behavior, and decrease in anxiety of mice is due to the activation of D2 dopamine receptors in the striatum after ICV ouabain injection, we performed a two-factor analysis of animal behavior under combined or separate administration of ouabain (50 µM, 0.5 µl bilaterally) and haloperidol (70 µg/kg intraperitoneally). Mice were habituated and separated into four equal groups with similar basal locomotor activity (8 animals per group): control group was administered 0.9% NaCl (intraperitoneally, 400 µl) 30 min prior to the ICV injection and aCSF (ICV, 0.5 µl bilaterally); ouabain-treated group was administered 0.9% NaCl (intraperitoneally, 400 µl) 30 min prior to the ICV injection and 50 µM ouabain in aCSF (ICV, 0.5 µl bilaterally); haloperidol-treated group was administered 5 µg/ml haloperidol in 0.9% NaCl (intraperitoneally, 70 µg/kg) 30 min prior to the ICV injection and aCSF (ICV, 0.5 µl bilaterally); haloperidol + ouabain-treated group was administered 5 µg/ml haloperidol in 0.9% NaCl (intraperitoneally, 70 µg/kg) 30 min prior to the ICV injection and 50 µM ouabain in aCSF (ICV, 0.5 µl bilaterally). After ICV injections the animals were tested in the open field for 20 min.

**Figure 3:**
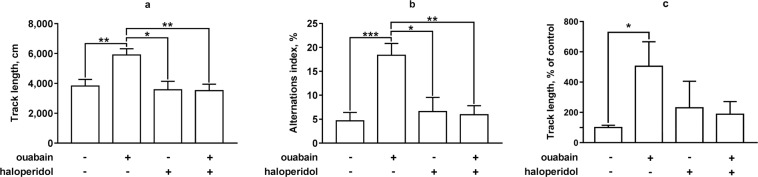
The influence of haloperidol on ouabain-induced hyperactivity, stereotyped movements, and anxiety in mice. Locomotor activity and stereotypic behavior of mice under combined or separate administration of ouabain (ICV, 50 µM, 0.5 µl bilaterally) and haloperidol (intraperitoneal, 70 µg/kg, 30 min prior to the ICV injection) in the open field test was assessed by total distance walked (**a**), alternations index (**b**), and percent of time spent in the center of the open field (**c**) respectively during a 20-min open field test. Results are presented as mean ± SEM, N = 8 per group; *p < 0.05, **p < 0.01, ***p < 0.001 according to two-way ANOVA with Tukey’s multiple comparisons test.

In the 20-min open field test locomotor activity of the animals from ouabain-treated group was 1.54 times higher than that in the control group (p = 0.0074, q = 5.003) (Fig. 3a). There was no difference in total locomotor activity between the control group and haloperidol-treated group (p = 0.9877, q = 0.4624), or haloperidol + ouabain-treated group (p = 0.9602, q = 0.6962) (Fig. 3a). Thus, ouabain increased locomotor activity in a 20-min open field test in the same way as in a 1 h open field test. Haloperidol at the dose used did not have any effect on the locomotor activity of mice, but completely attenuated the effect of ouabain (p = 0.0070, q = 5.035).

Stereotypic behavior of all groups was assessed using the alterations index in a 20-min open field test. Alternations index in ouabain-treated group was 3.93 times higher than that in the control group (18.36 ± 2.46% and 4.66 ± 1.49%) (p = 0.0002, q = 7.135) (Fig. 3b). There was no significant difference between the control group and haloperidol-treated group (4.02 ± 1.81%) (p = 0.9560, q = 0.7213) or haloperidol + ouabain-treated group (5.94 ± 1.87%) (p = 0.9997, q = 0.1262) (Fig. 3b). Thus, ouabain increased stereotypic behavior in a 20-min open field test, while haloperidol at the dose used did not affect the stereotypic behavior of mice, and attenuated effect of ouabain on the alternations index (p = 0.0017, q = 5.85), compared to the ouabain-treated group).

The degree of anxiety for all four groups was evaluated through a comparison of the length of the track segment travelled in the central zone relative to the control group. The ouabain-treated group travelled 505.1 ± 161.6% in the center relative to the control (p = 0.0335, q = 4.125) (Fig. 3c). Haloperidol in 70 µg/kg did not have an influence on the length of the track segment spent by animals in the center of the open field (p = 0.8876, q = 1.020) (Fig. 3c), while at the same time preventing an ouabain-induced increase. The track length in the central zone of the haloperidol + ouabain-treated group did not differ significantly from that of the control (p = 0.9347, q = 0.8322) (Fig. 3c). Taken together, haloperidol prevented the influence of ouabain on the locomotor activity of animals, manifestations of stereotypic behavior in the open field test, and ouabain-induced decrease in anxiety.

### Ouabain influence on dopamine extracellular concentration and reuptake

**Figure 4:**
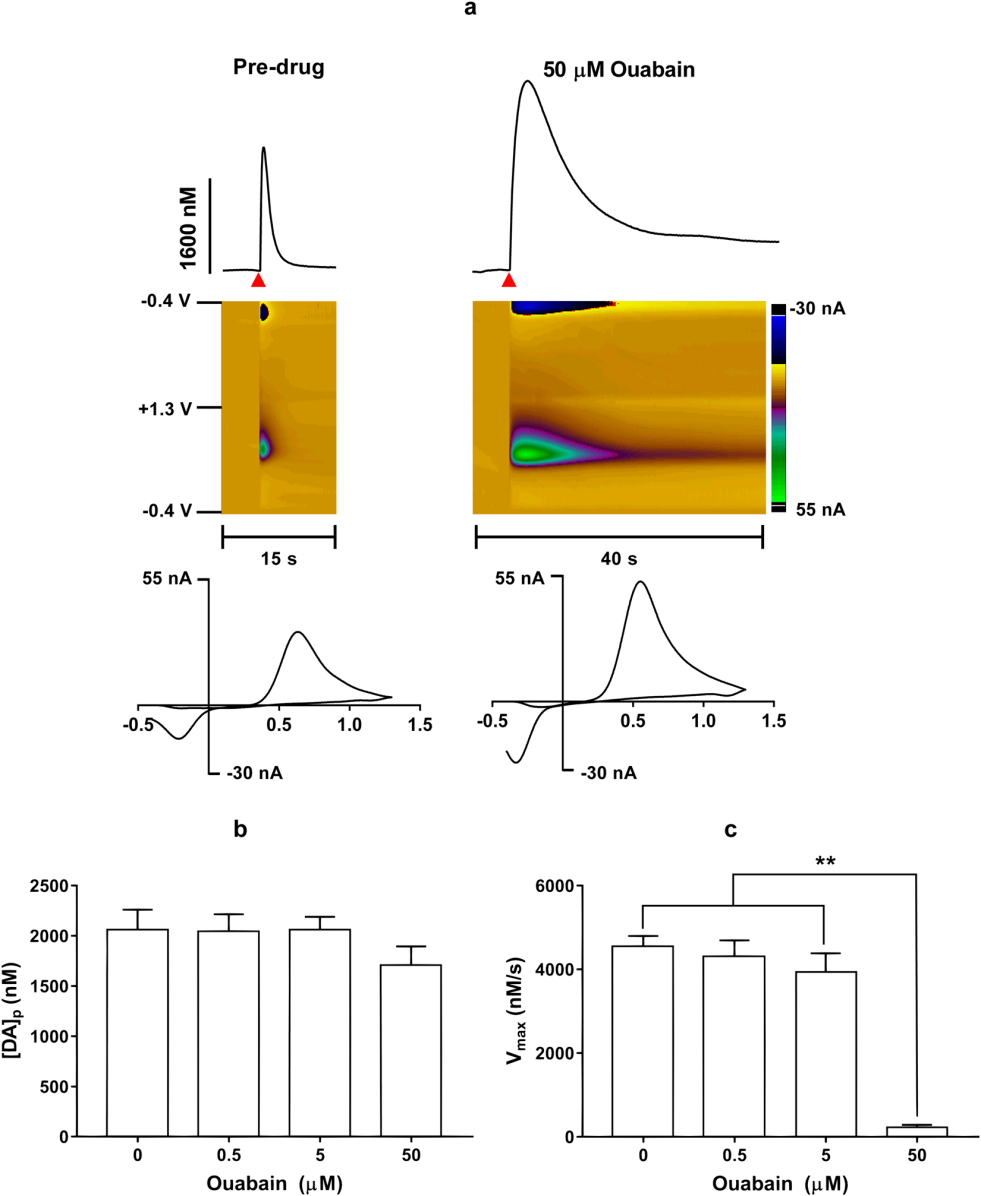
(**a**) Representative traces (top) with their respective false color plots (middle) and voltammograms (bottom) of electrically-evoked DA in the mouse dorsolateral striatum taken before (left) and 20 min after (right) ouabain (50 μM) perfusion. These signals had oxidation and reduction peaks at +0.6 V and −0.2 V, respectively, identifying the measured species as DA. The false color plots depict the voltammetric data, with time on the *x*-axis, applied scan potential on the *y*-axis, and background-subtracted faradaic current shown on the *z*-axis in pseudo-color. The red triangle indicates an electrical stimulation. Twenty minutes after administration, ouabain did not have a significant effect on DA release per stimulus pulse at any concentration (**b**) but did significantly reduce DA uptake (**c**), measured as the *V*_*max*_, at the highest concentration (50 μM) but not lower concentrations (0.5 μM and 5 μM). Data are presented as means ± SEM of 5 mice. **p < 0.01.

We have proposed that observed effects of ouabain are due to an increase in extracellular dopamine concentration. In order to determine the effects of ouabain on DA dynamics, brain slices containing the dorsal striatum were taken from naïve mice and used for fast-scan cyclic voltammetry (FSCV) experiments. Electrically-evoked DA efflux was measured before and after the exposure to increasing concentrations (0.5 μM, 5 μM, and 50 μM) of ouabain *in vitro* (for representative plots see Fig. 4a).

Ouabain at a concentration of 50 μM dramatically decreased DA uptake rate in the mouse dorsolateral striatum as measured by *in vitro* fast-scan cyclic voltammetry. A repeated measures one-way ANOVA found that DA release per stimulus pulse was not significantly different between the different concentrations (F(1.334, 5.335) = 1.85; *p* = 0.237; Fig. 4b). The uptake of DA, measured as the *V*_*max*_, was also analyzed using a repeated measures one-way ANOVA and showed that DA uptake rate, was significantly different between concentrations (F(1.391,5.563) = 92.11; *p* < 0.0001). A post hoc Tukey’s multiple comparisons test found that the highest concentration of ouabain (50 μM) significantly decreased DA uptake compared to the pre-drug baseline and lower concentrations (0.5 μM and 5 μM) of ouabain (Fig. 4c).

### Dopamine metabolites content in the striatum after ICV ouabain administration

In order to determine if the ouabain-induced hyperactivity, stereotypy, and reduced anxiety observed were associated with an increase in the extracellular concentration of dopamine or dopamine synthesis, we measured the content of dopamine (DA), 3,4-dihydroxyphenylacetic acid (DOPAC), and homovanillic acid (HVA), in the striatal tissues of control and ouabain mice 1 h after the ICV injection.

**Figure 5:**
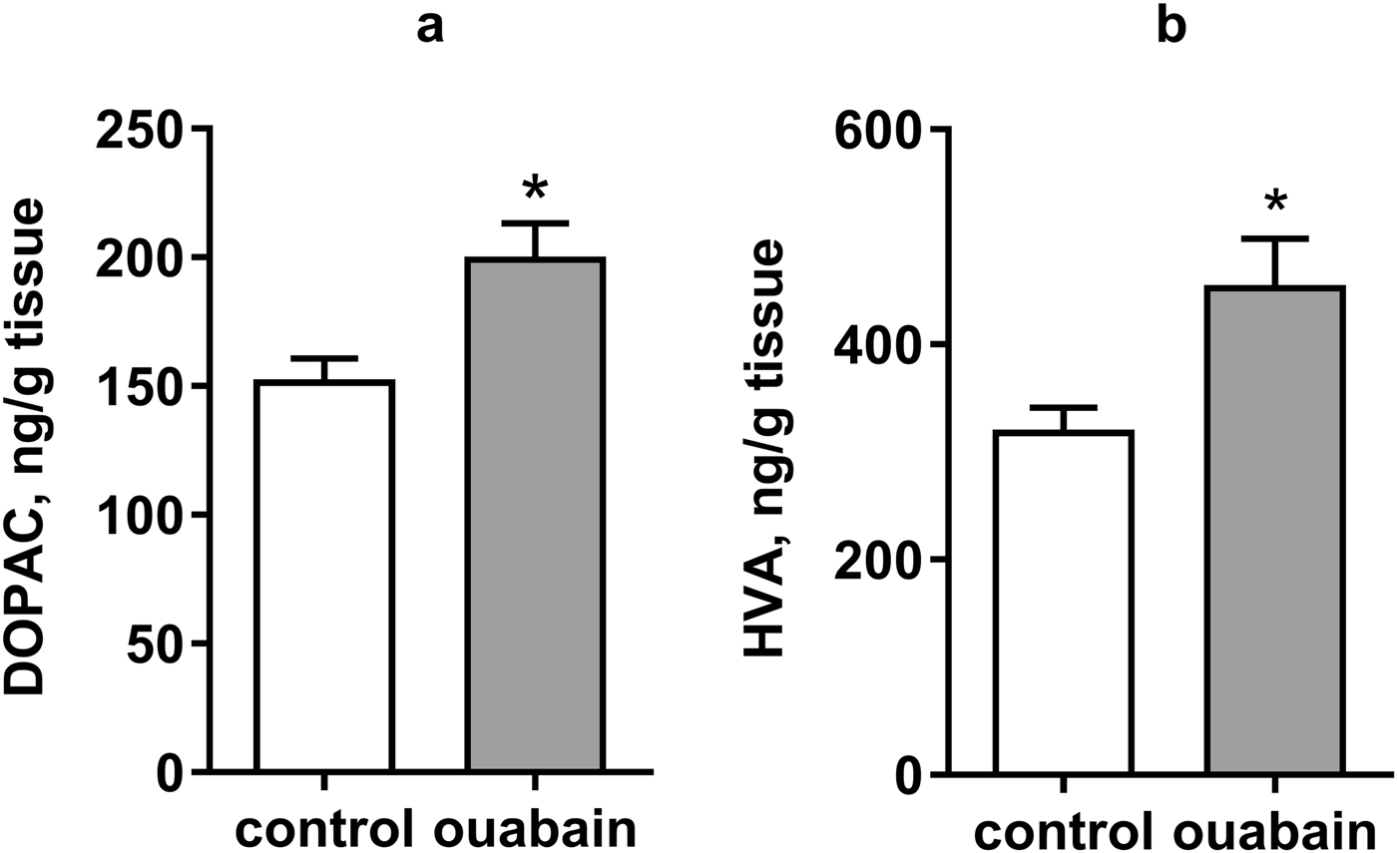
The effect of ouabain on the content of dopamine metabolites in the striatum of mice. The content of 3,4-dihydroxyphenylacetic acid (DOPAC) (**a**) and homovanillic acid (HVA) (**b**) in striatal tissue samples of control and ouabain-treated (ICV, 50 µM, 0.5 µl bilaterally) mice 1 h after the ICV injection. Results are presented as mean ± SEM, N = 8 per group; *p < 0.05 compared to control according to unpaired t-test.

The level of dopamine in striatal tissues did not differ significantly between the control and ouabain-treated groups 1 hr after the ICV injection (see Supplementary Fig. S7). Evaluation of the level of catecholamines in striatal tissues, meanwhile, showed an increase in DOPAC concentration by 1.32 times (p = 0,0136, t = 2.707) and that of HVA increased by 1.42 times (p = 0.0224, t = 2.474) compared to the control group (Fig. 5). The rise in HVA indicates a reduced uptake rate of dopamine from the synaptic cleft. The stable dopamine level, combined with the increase in DOPAC level, suggests that re-synthesis of dopamine in tissues is enough to compensate for the reduced uptake caused by ouabain administration.

Bearing in mind the ouabain-induced increase in the dopamine metabolites level of striatal tissue without any changes in dopamine content, supported by a reduction of uptake rate demonstrated by fast-scan cyclic voltammetry *in vitro*, we can conclude that observed effects of ouabain are associated with an increase in extracellular dopamine level as consequence of reduced uptake rate, and resulting compensational dopamine synthesis.

### Akt, GSK3β and ERK1/2 activation in the striatal tissue of the experimental animals

To determine the influence of D2 dopamine receptor activation on ouabain-induced intracellular signal cascades in the striatum of mice, Akt, GSK3β and ERK1/2 activation was evaluated 30 min after the bilateral ICV injection of 0.5 μl of 50 μM ouabain (i.e. 1 h after intraperitoneal administration of haloperidol, 70 µg/kg).

**Figure 6:**
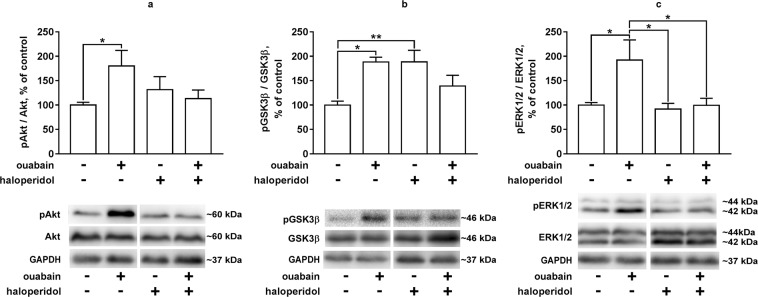
Akt, GSK3β and ERK1/2 activation in the striatum of mice under combined or separate administration of ouabain and haloperidol. Akt (**a**), GSK3β (**b**) and ERK1/2 (**c**) phosphorylation in the striatal tissue of mice under combined or separate administration of ouabain (ICV, 50 µM, 0.5 µl bilaterally) and haloperidol (intraperitoneal, 70 µg/kg, 30 min prior to the ICV injection) 30 min after ouabain administration. Results are presented as mean ± SEM, N = 8 per group; **p < 0.01, ***p < 0.001 according to two-way ANOVA with Tukey’s multiple comparisons test. Representative bands are combined: control + ouabain and haloperidol + ouabain-haloperidol (for full set of the bands see Supplementary Fig. S8).

The activation of Akt in ouabain-treated group increased (phosphorylation increased) by 1.8 times compared to the control group (p = 0.0299, q = 4.22). At the same time, no significant changes in Akt phosphorylation were observed in the striatum of mice while comparing other groups: control group vs haloperidol-treated group, (p = 0.6467, q = 1.664); control group vs haloperidol + ouabain-treated group, (p = 0.9552, q = 0.7257); ouabain-treated group vs haloperidol + ouabain-treated group, (p = 0.1214, q = 3.274) (Fig. 6a).

The activation of GSK3β in ouabain-treated group decreased (phosphorylation increased) by 1.88 times compared to the control group (p = 0.0149, q = 4.632). The administration of haloperidol also caused a 1.88-time increase in the phosphorylation of GSK3β (p = 0.0077, q = 5.015) (Fig. 6b). However, in the haloperidol + ouabain-treated group, no significant difference in GSK3β phosphorylation was observed compared to the control (p = 0.4157, q = 2.212) or ouabain-treated (p = 0.2840, q = 2.583) groups (Fig. 6b).

There was a 1.8-fold increase in the activation of ERK1/2 (increased phosphorylation) in the ouabain-treated group compared to the control group (p = 0.259, q = 4.36) (Fig. 6c). At the same time, in the striatum of haloperidol-treated and haloperidol + ouabain-treated mice, there were no significant changes in ERK1/2 phosphorylation compared to the control group (p = 0.9919, q = 0.3999 and p > 0.9999, q = 0.02306, respectively) (Fig. 6c). Haloperidol attenuated ERK1/2 activation in the striatal tissue of ouabain-treated mice (p = 0.0320, q = 4.224).

Thus, ICV ouabain administration caused activation of Akt, deactivation of GSK3β and activation of ERK1/2 in the striatum of mice. Haloperidol administration attenuated these effects of ouabain.

### Histological analysis of brain structures after ouabain administration

Histological analysis was performed in order to evaluate whether ouabain effects described above are associated with neurotoxicity. Morphometric analysis revealed no significant changes in neuronal density in analyzed regions of striatum, CA1 and CA3 areas of hippocampus, and motor cortex of ouabain-treated mice compared to the control ones 24 h after the ICV injection. There was no significant difference in the percentage of neurons with dystrophic changes in the striatum, CA1 and CA3 areas of hippocampus, and motor cortex of ouabain-treated mice compared to the control ones (Table 1).

**Table 1:** Neuronal density (number of neurons per 1000 μm^2^) and the percentage of dystrophically altered neurons in the striatum, hippocampus and motor cortex of mice from the control group and ouabain-treated group (25 pmol ouabain per lateral ventricle).

	Control	Ouabain-treated, 25 pmol/ventricle	T-test, р
**Striatum (N = 10 per group)**			
neurons/1000 µm^2^	1.78 ± 0.06	1.69 ± 0.05	0.31 (ns)
neurons with dystrophic changes, %	10.36 ± 1.69	12.68 ± 2.12	0.41 (ns)
**Hippocampus CA1 (N = 10 per group)**			
neurons/1000 µm^2^	7.35 ± 0.10	7.29 ± 0.12	0.074 (ns)
neurons with dystrophic changes, %	12.13 ± 1.04	14.14 ± 1.30	0.24 (ns)
**Hippocampus CA3 (N = 10 per group)**			
neurons/1000 µm^2^	4.33 ± 0.08	4.26 ± 0.07	0.56 (ns)
neurons with dystrophic changes, %	12.27 ± 1.27	13.98 ± 1.70	0.43 (ns)
**Motor cortex (N = 10 per group)**			
neurons/1000 µm^2^	1.98 ± 0.05	1.92 ± 0.06	0.43 (ns)
neurons with dystrophic changes, %	34.96 ± 4.74	26.45 ± 4.09	0.19 (ns)

Results are presented as mean ± SEM, N = 10 per group.

**Figure 7:**
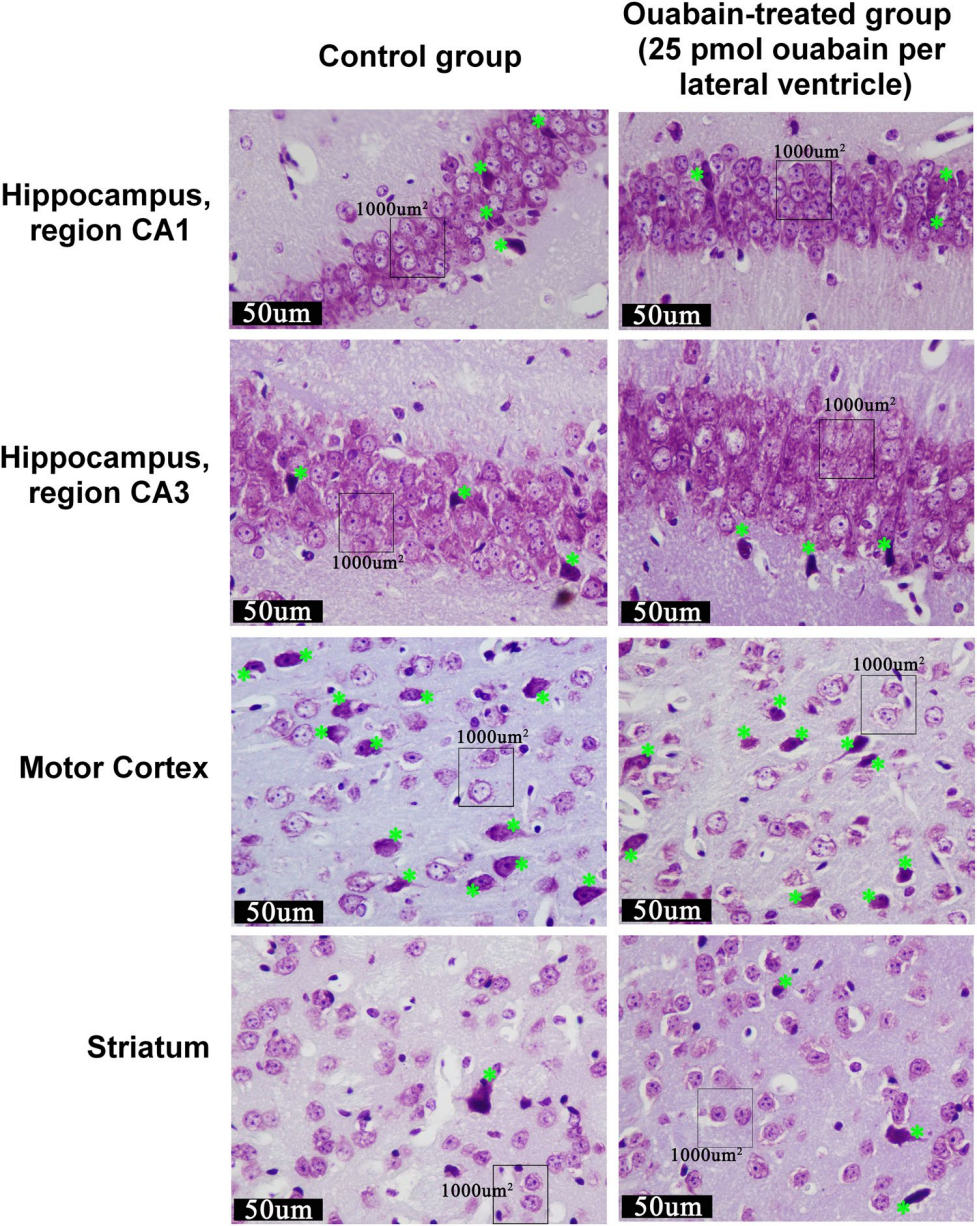
Microphotographs of the striatum, hippocampus and motor cortex of mice from the control group and ouabain-treated group (25 pmol ouabain per lateral ventricle). Nissl staining, distrophically altered neurons are labelled by green stars.

Histological analysis of striatum tissue did not show any structural damage; neurons and tissues of the ouabain-treated group did not visually differ from the control group (Fig. 7).

Histological analysis of the motor cortex and hippocampus of both control and ouabain-treated mice revealed defects in brain tissue near cannulas position caused by mechanical damage to the tissue through the surgery and injection procedure. Damage to the tissues and neurons of the motor cortex and hippocampus was similar in both the ouabain-treated and the control groups.(see Supplementary Fig. S3).

## Discussion

In this report, we describe a novel model of mania-like behavior in mice, induced by acute ICV ouabain administration, similar to the existing model in rats. However, the dose of ouabain which induced hyperactivity, 0.5 µl 50 µM (25 pmol, 14.6 ng) for each lateral ventricle of the brain, was lower for mice than that for rats (5 µl 1 mM)^15,16,21^. This is likely due to the smaller size of the mouse brain. In fact, higher doses of ouabain tested led to a decrease in locomotor activity of mice (see Supplementary Fig. S4), which is consistent with previous observations^37^. The hyperactivity, stereotypy, and decreased anxiety in ouabain-treated mice is already observed during the first 10 min of the open field test immediately after ouabain injection. It is also worth noting that the locomotor activity decreases with time in both groups, however, a significant difference between the groups persists throughout the 1 h test. The total distance covered in the open field by mice in the ouabain-treated group was 1.5 times greater than that in the control group, which is similar to the results obtained previously in rats^15,16,21^. Ouabain-treated mice also demonstrated stereotyped movements and decreased anxiety levels in the open field, in contrast to the control animals. These effects were relatively short-lived and 3 h after ouabain administration were no longer observed.

It can be hypothesized that both hyperactivity and stereotypy of ouabain-treated mice are associated with hyperactivation of the dopaminergic system, the same way they are observed in mice following methamphetamine or cocaine administration^38^ or in DAT-KO mice^39,40^. It is known that hyperactivation of the D2 dopamine receptor leads to hyperlocomotion and ERK1/2 activation in mice after cocaine administration^41^. Therefore, we tested if observed behavioral alterations induced by ouabain were realized through D2 dopamine receptor activation, and performed two-factor analysis using selective D2 receptor antagonist haloperidol. Administered at a low dose, haloperidol alone did not affect locomotor activity, stereotypic behavior, or anxiety levels of drug-naïve mice. It did, however, attenuate ouabain-induced mania-like behavior, like it did in mice administered methamphetamine^38^.

In the rat model, increased locomotor activity^16^ and cognitive flexibility impairment^24^ were demonstrated as characteristics reflecting the mania-like phase. In a recent study, both the mania-like and the depressive phase were demonstrated in rats, along with the accompanying oxidative stress and Na, K-ATPase dysfunction, the same way as they appear in patients with bipolar disorder^23^. In our mice model of ouabain-induced mania, the mania-like phase is illustrated with hyperlocomotion, stereotyped behavior, and a lessened degree of anxiety.

Since hyperactivity in mice is associated with dopamine receptor activation, we assumed that it may be due to increased extracellular concentration of dopamine caused by impaired dopamine reuptake^42^, like it is, for example, in DAT-KO mice^43^.

Data procured using fast-scan cyclic voltammetry showed a drastic drop in dopamine uptake rate in response to 50 µM ouabain administration, as mentioned in previous studies^20^, leading to an increased concentration in the synaptic cleft, and, as consequence, hyperactivation of the dopamine receptors. This confirms that the observed hyperactivity in mice detected during the first hour after ouabain administration is caused by a decreased reuptake of dopamine. This data are consistent with previous studies showing that ouabain administered to both mice and rats causes an increase in the extracellular dopamine level^19,20,44^.

The observed increase in the striatal tissue content of dopamine metabolite HVA also indicates that reuptake is decreased, while the increase in DOPAC in conjunction with a stable level of dopamine confirms an increase in synthesis, as was shown in previous studies in the rat model^32^.

If dopamine is retained for a longer period of time in the synaptic cleft, hyperactivation of dopamine receptors occurs, which, as shown by us using a two-factor analysis with haloperidol administration, leads to mania-like behavior in the mice model. In turn, previous studies in different models with hyperactivation of D2 receptors showed different mechanisms of how the response is mediated on the level of intracellular signaling cascades.

It was previously described that there are two different ways for the activation of D2 dopamine receptor to affect the Akt/GSK3β pathway in the striatum. One is a short-acting pathway (<30 min) mediated by G proteins, followed by PI3K activation or involvement of BDNF, Akt activation and GSK3β deactivation. The other one is a long-acting pathway through the activation of D2 dopamine receptor/β-arrestin/PP2A complex, leading to Akt deactivation and subsequent GSK3β activation^45^. We showed that 30 min after the ICV injection ouabain caused activation of Akt and deactivation of GSK3β in the striatum, suggesting the activation of the short-acting G protein-mediated signaling pathway rather than the β-arrestin-mediated one. In the existing model of mania in rats, it was also shown that 30 min after the ICV injection of ouabain, Akt activation and GSK3β deactivation occur^16^. Previously it was shown that acute (up to 30 min) cocaine administration activates signaling cascades through G protein-dependent pathway caused by the activation of D2 dopamine receptor in mice^46^, which is consistent with our results. In our experiments, haloperidol administration also caused GSK3β deactivation which is consistent with literature data^47^. In addition, the activation of ERK1/2, characteristic of the activation of dopamine receptors^48^, was shown. These effects of ouabain were not observed in the striatum of mice pre-treated with haloperidol, indicating the dependence of these effects on the activation of D2 dopamine receptors.

Since high concentrations of ouabain can be neurotoxic^14,49^, the observed decrease in the activity of animals might have been potentially associated with neuronal death. To resolve this question, we conducted a histological analysis, which showed that ouabain administration does not cause neuronal death in the striatum, hippocampus, and motor cortex of the experimental animals. This allows for further investigation of the effects of ouabain administration in this model, without them being caused by neurotoxicity of ouabain in the administered dosage.

We managed to replicate previously described effects of ouabain on hyperactivity in mice, and we discovered that ouabain causes an increase in stereotypical behavior and a lessened degree of anxiety, which replicated three aspects of mania-like behavior. Also, we showed that the effects are consequence of a decrease in dopamine uptake rate, and the following hyperactivation of D2 receptors. In our model, the short-term effects of ouabain administration were investigated both on the behavioural manifestations, and the level of neurotransmitters and intracellular signaling cascades. Long-term ouabain-induced changes, detailed investigation of how functional activity of the Na, K,-ATPase is linked to these processes, and the assessment of both the mania and depressive phases call for further research on the mice model established in the current publication.

## Methods

### Animals

The mice were kept in compliance with the rules for using laboratory animals in scientific research (according to the recommendations of the Federation for Laboratory Animal Science Associations (FELASA) and Russian Laboratory Animal Science Association (Rus-LASA)). 4–6-month-old male mice (line C57Bl/6) from Saint Petersburg State University vivarium with individually ventilated cages were used in the experiments. Animals were maintained at a temperature of 22 ± 1 °C, 50–70% relative humidity and a 12 h light/dark cycle (light from 8 a.m. to 8 p.m.), food and water ad libitum. The experiments were carried out in accordance with European guidelines on animal experimentation and approved by the Ethical Committee for Animal Experiments of Saint Petersburg State University, permission #131-03-1.

### Experimental groups

The total number of mice in the experiments performed to acquire presented data was 73. 20 mice (10 in the control group and 10 in ouabain-treated group) were used for 1 h open field tests before the injection, immediately after the injection, 3 h and 24 h after the injection, as well as for histological analysis. 32 mice (8 in the control group, 8 in ouabain-treated group, 8 in haloperidol-treated group, and 8 in haloperidol + ouabain-treated group) were used for 20-min open field tests and western blotting. 16 mice (8 in the control group and 8 in ouabain-treated group) were used for determination of the content of dopamine metabolites. 5 mice were used for fast-scan cyclic voltammetry evaluation of dopamine uptake rate.

### Surgery procedures

Surgery was performed under Zoletil (Virbac Sante Animale, France) anesthesia (intraperitoneal injections of 200 mg/kg) with intramuscular injection of Rometar (Bioveta, Czech Republic) (0.1 mg/kg). The skull was carefully cleaned and dried.

Guide cannulas were made of 26 g needles (KDF, Germany) and 1 × 2 mm fixed plastic holders, dummy and injection cannulas were made of 33 g needles (Mesoram, Italy). Two guide cannulas were placed bilaterally: AP = −0.5; L = 1.0; depth 2.0; (coordinates are given in millimeters relative to the bregma^50^) and fixed with dental acrylic cement. After cement consolidation, two dummy cannulas were inserted into the guide cannulas and mice were kept in the maintaining room for 2 days before experimental procedures.

### Drug administration

ICV injections were performed by inserting injection cannulas into the guide cannulas 2.5 mm deep following a 2-day rehabilitation period after surgery. Injection cannulas were connected to Hamilton syringes by polyethylene tubing (i.d. 0.28 mm). 0.5 μl of artificial cerebrospinal fluid (aCSF: 125 mM NaCl, 26 mM NaHCO_3_, 4 mM KCl, 1.25 mM NaH_2_PO_4_, 2 mM CaCl_2_, 2 mM MgCl_2_, 25 mM glucose) or 50 µM ouabain (Sigma, USA) in aCSF was injected into the left and right ventricles for 2 min at a speed of 0.5 μl/min using a syringe pump (0.5 μl in each ventricle). After injections, mice were immediately tested in the open field. In the haloperidol and haloperidol + ouabain-treated groups of animals 5 µg/ml haloperidol (Sigma, USA) in 0.9% NaCl (intraperitoneally, 70 µg/kg) was administered 30 min before the ICV injections.

### Open field test

The open field test was used to assess spontaneous locomotor activity and stereotypical behavior in mice. The apparatus (square 40 × 40 cm box, Open Science, Russia) was divided into nine zones: four “corner” zones (10 × 10 cm), four “wall” zones (10 × 20 cm) and one “center” zone. Each mouse was placed individually on the center of the arena and a 1 h habituation in the open field was performed before the ICV injection. Then open field tests were performed for 1 h immediately after the injection, 3 h and 24 h after the injection. Animal behavior was monitored by the video tracking system (EthoVision XT video tracking software, Noldus), a video camera was placed above the box in a uniformly lit room. Total distance traveled by mice was calculated. In analyzing the stereotypical behavior, we applied an existing methodology for assessing the stereotypes of motor behavior in the open field^51^, modified using the program EthoVision XT. To evaluate stereotypical behavior in mice the spontaneous alternations index was calculated using EthoVision tool for analysis of stereotypical behavior. The number of alternations (*Alternations*) was calculated as the number of trajectory segments in which the animal visited all selected adjacent zones of the open field consecutively (except for the central zone). Thus, the number of alternations is equal to the total number of complete circular trajectories. The maximum possible number of alternations (*Max alternations*) was calculated as the number of zones selected for analysis minus one zone, subtracted from the total number of erratic visitations of all of the selected zones. The *Alternations index* was calculated as *Alternations*/*Max alternations* × 100. Since the *Alternations index* is a relative value, it reflects stereotypical behavior regardless of the locomotor activity, which affects both the number of alternations and the maximum possible number of alternations. Statistical analysis of the data was performed using GraphPad Prism 7 software. Data analysis for multiple groups with two variables was performed using Shapiro-Wilk normality test and two-way ANOVA, p value was calculated using Tukey’s multiple comparisons test.

### Elevated plus maze

The elevated plus maze test was performed in a 65 × 65 × 40 cm elevated plus maze (Open Science, Russia) in a uniformly lit room for 5 min immediately after the ICV injections. Time spent and in open and closed arms of the maze were calculated using EthoVision XT video tracking software (Noldus). The number of head-dips was calculated manually. Statistical analysis of the data was performed with GraphPad Prism 7 software using Shapiro-Wilk normality test, p value was calculated using unpaired t-test.

### Fast-scan cyclic voltammetry (FSCV)

The mice were decapitated after administration of isoflurane anesthesia. The brain was promptly removed and immediately submerged in oxygenated ice-cold artificial cerebrospinal fluid (aCSF): 126 mM NaCl, 2.5 mM KCl, 1.2 mM NaH2PO4 (monobasic), 2.4 mM CaCl2, 1.2 mM MgCl2, 0.4 mM L-ascorbic acid, 11 mM C6H12O6, 25 mM NaHCO3, pH 7.40. A vibrating tissue slicer (Vibratome 1000 Plus, The Vibratome Company, St. Louis, MO, USA) was used to obtain coronal slices (400 µm thick) containing the dorsal striatum. The slices were then placed in oxygenated aCSF at room temperature, and equilibrated for at least 30 minutes, after which they were placed on a submersion recording chamber in which oxygenated aCSF was flowing at a rate of 1 mL/min at room temperature. With accordance to previous studies^52–54^ carbon fiber microelectrodes (diameter of fiber: 7 µm; Goodfellow Cambridge Ltd., Huntington, UK) were assembled and connected to a voltammetric amplifier (UNC Electronics Design Facility, Chapel Hill, NC, USA). The carbon fiber microelectrode was then placed into the dorsolateral striatum. A twisted bipolar stimulating electrode (Plastics One, Roanoke, VA, USA), connected to a voltage output box, was placed on the tissue surface next to the recording elctrode at a distance of approximately 200 µm from it. Every ten minutes, electrical, singular, rectangular pulses (monophasic, 350 µA, 4 ms/phase) were used to induce DA release. Every 100 ms for at least 15 s, extracellular DA was recorded at the carbon fiber microelectrode via application of a a triangular waveform (from −0.4 V to +1.3 V and back to −0.4 V vs Ag/AgCl, 400 V/s).

Observation of background-subtracted cyclic voltammograms was used to identify DA. Oxidation and reduction peaks occurring at ~ +0.6 and ~−0.2 V, respectively (vs. Ag/AgCl reference) were the characterizing aspect. Once a stable baseline DA signal was acquired (3 recordings within 10% variability), ouabain in increasing concentrations (0.5 µM, 5 µM, and 50 µM ouabain in oxygenated aCSF) was washed over the slice over the course of 20 minutes for each concentration. A computer was used to store the digitized data (National Instruments, Austin, TX, USA). After each experiment, carbon fiber microelectrode response was calibrated using a flow injection analysis system. A known concentration of DA (1 μM, Sigma Aldrich, St. Louis, MO, USA) dissolved in a calibration buffer with pH 7.4 at room temperature was used in triplicate to perform the calibrations.

FSCV kinetic analysis. DA release parameters (i.e., DA release per stimulus pulse ([DA]p) and uptake (Vmax)) of recordings prior to the drug administration were calculated using kinetic modeling (LVIT software, UNC, Chapel Hill, NC, USA)^52,55,56^. These parameters were modeled assuming to follow Michaelis-Menten kinetics. DA concentration changes with respect to time were estimated by equation:

$${\rm{d}}[{\rm{DA}}]/{\rm{dt}}=({\rm{f}})\,{[{\rm{DA}}]}_{{\rm{p}}}-{(V}_{{\rm{\max }}}/({\rm{Km}}/[{\rm{DA}}])+{\rm{1}}))$$

where f is the stimulation frequency (Hz), [DA]p is the concentration of DA released per stimulus pulse, and Vmax and Km are Michaelis-Menten rate constants for DA uptake. The baseline value of Km was set to 0.16–0.20 μM^55,56^.

Data was analyzed using GraphPad Prism (GraphPad Software version 7.04, San Diego, CA, USA). Repeated measures one-way ANOVAs and Tukey’s multiple comparisons tests (where appropriate) were used to analyze DA release and uptake data. Data is presented as mean ± SEM and the criterion for significance was set at p < 0.05.

### Determination of catecholamine levels

Cervical dislocation was applied to mice, followed by decapitation. The brain was removed on ice and the striatum was isolated and homogenized in 20 volumes of extraction medium (0.1 N HClO_4_ with 0.25 nmol/ml DBS (3,4-dihydroxybenzylamine) added as an internal standard) using a glass/Teflon pestle homogenizer (0.2 mm) Shuett Homgen plus (SchuetBiotec GmbH, Germany) at a pestle rotation speed of 3,000 rpm in ice water bath. Samples were centrifuged in a refrigerated centrifuge at 10,000 g for 15 min (t = 4 °C). The content of monoamines and their metabolites was determined in the supernatant using high performance liquid chromatography (ion-pair chromatography) with electrochemical detection (HPLC-ED) on a System Gold liquid chromatograph (Beckman Coulter, Inc., USA) equipped with a Rheodyne 7125 injector (USA) with a 20 μl sample loop. The studied substances were separated on a reverse-phase column Nucleodur C18 Gravity, 4.6 × 250 mm, pore diameter of 5 μm (Mashery-Nagel GmbH & Co. KG, Germany). Mobile phase (0.1 M citrate-phosphate buffer (pH 3.0) containing 1.1 mM octanesulfonic acid, 0.1 mM EDTA and 9% acetonitrile) flow rate of 1 ml/min at a pressure of 200 atm was achieved using a System Gold 125 pump (Beckman Coulter, Inc., USA). The measurements were performed using an EC3000 electrochemical detector (RECIPE Chemicals + Instruments GmbH, Germany) equipped with a ClinLab ECD cell, Model Sputnik, with a glassy carbon working electrode (+0.85 V) and an Ag/AgCl reference electrode. Sample registration and chromatogram processing were performed using MULTICHROM 1.5 software (AMPERSAND, Russia). All reagents used for the analysis were of analytical grade. A mixture of working standards of the detected substances at a concentration of 0.25 nmol/ml was used for calibration of the chromatograph. The concentration of monoamines in the test samples was determined using the method of internal standard, by calculating the ratio of the peak areas in the standard mixture and in the test sample^57^. Statistical analysis of the data was performed with GraphPad Prism 7 software using Shapiro-Wilk normality test, p value was calculated using unpaired t-test.

### Western blot

After the behavioral tests described above (groups with a 20-min open field test), the striatum was isolated from the brain of mice 30 min after ouabain injection. The brain tissue samples were lysed in RIPA buffer (Sigma, USA) containing cocktails of protease and phosphatase inhibitors (Sigma, USA). The lysate was centrifuged at 14,000 g for 20 min, then the supernatant was collected. The protein concentration in the samples was measured using DC Protein Assay Kit (Bio-Rad, USA). Proteins were separated using Laemmli polyacrylamide gel electrophoresis, transferred to Westran Clear Signal (Whatman, United Kingdom) PVDF membranes, and stained with antibodies according to manufacturer recommendations. Primary antibodies to Akt, p-Akt (Ser473), ERK1/2, GSK3, p-GSK3 (Ser9) (Cell Signaling Technology, USA), p-ERK1/2 (Thr202/Tyr204), GAPDH and β-actin (Santa Cruz Biotechnology, USA) and secondary anti-rabbit and anti-mouse antibodies (Cell Signaling Technology, USA) conjugated with horseradish peroxidase were used. The membranes were developed using Super Signal West Femto Maximum Sensitivity Substrate or Super Signal West Pico Chemiluminescent Substrate (Thermo Scientific, USA). The luminescence of the bands was detected using ChemiDoc XRS + (Bio-Rad, USA) gel documentation system, the intensity was calculated using Image Lab 3.0 software (Bio-Rad, USA). Kinase activation was assessed by the level of phosphorylation, i.e. the ratio of the intensity of bands of the phosphorylated form of the kinase to the intensity of bands of its total form. Evaluation of the kinases total form alterations was made by the ratio of the intensity of bands of the kinases total form to β-actin or GAPDH. Statistical analysis of the data was performed using GraphPad Prism 7 software. Data analyses of multiple groups with two variables was performed using Shapiro-Wilk normality test and two-way ANOVA, p value was calculated using Tukey’s multiple comparisons test.

### Histochemical analysis

10 mice in the control group and 10 mice in ouabain-treated group were used in a morphological study of the brain regions. After fixation in a 4% PFA solution in a phosphate buffer (pH 7.4), the brain was cut into 6 2-mm-thick frontal slices, dehydrated in alcohols of increasing concentration and embedded in paraffin. From each 2-mm-thick slice, 4 5-μm-thick slices were made, 1 of which was stained with hematoxylin and eosin for histological analysis, while the other 3 were stained with 0.1% cresyl violet aqueous solution (Nissl staining) for counting neurons in the motor cortex, striatum and pyramidal layer of the hippocampus. Analysis of the motor cortex (MOs, MOp) was carried out in the frontal slices at the level of 0.145‒1.845 mm, striatum (CP) − 0.145‒1.245 mm, and the pyramidal layer of the hippocampus (CA1, CA3) − (−1.455)‒(−2.78) mm from the bregma^50^.

The number of neurons was counted in photographs taken with an Eclipse 50i microscope (Nikon, Japan) equipped with a DS-Fi1 digital camera (Nikon, Japan) using a 40× objective (resolution 0.25 µm). In each image, the total number of neurons was calculated as well as the number of neurons with dystrophic changes, which included shriveled, pyknotic, deformed and hyperchromic neurons, and neurons with focal or total chromatolysis. In each of the analyzed areas, at least 500 neurons in 5–25 non-intersecting fields of view were counted in three sections (striatum in 5 fields of view with an area of 56,000 μm^2^, motor cortex in 6 fields of view with an area of 56,000 μm^2^, and zones of the hippocampus in 21–25 fields of view with an area of 3,700 μm^2^). Then, for each analyzed area of each mouse brain, the average number of neurons per 1,000 μm^2^ (for convenience of presentation) and the average percentage of neurons with dystrophic changes were determined. Statistical analysis of the data was performed using GraphPad Prism 7 software. Data analysis was performed using Shapiro-Wilk normality test, p value was calculated using unpaired t-test.

## Supplementary information

Supplementary Info

## Data Availability

Data supporting the findings of the current study are available from the corresponding author on reasonable request.
