# Supplementary material for: Intracerebroventricular injection of ouabain causes mania-like behavior in mice through D2 receptor activation
Source: Sci Rep. 2019 Oct 30;9:15627. doi: 10.1038/s41598-019-52058-z (PMC6821712; doi:10.1038/s41598-019-52058-z)
Supplement: Supplementary file 1 — Supplementary Info [file 41598_2019_52058_MOESM1_ESM.pdf]

Intracerebroventricular injection of ouabain causes mania-like behavior in mice through D2 receptor activation

Alexander Lopachev[1,*] (lopachev@neurology.ru), Anna Volnova[2,3] (a.volnova@spbu.ru), Anna Evdokimenko[1] (annevdokimenko@gmail.com), Denis Abaimov[1] (abaidenis@yandex.ru), Yulia Timoshina[4] (july.timoschina@yandex.ru), Rogneda Kazanskaya[3] (magnolia@kwakwa.org), Olga Lopacheva[1,5] (olga3511@yandex.ru), Alex Deal[6] (adeal@wakehealth.edu), Evgeny Budygin[6] (ebudygin@wakehealth.edu), Tatiana Fedorova[1] (tnf51@bk.ru), Raul Gainetdinov[2] (gainetdinov.raul@gmail.com)

[1] Research Center of Neurology, Volokolamskoye shosse 80, 125367 Moscow, Russia;
[2] Institute of Translational Biomedicine, Saint Petersburg State University, Universitetskaya Emb. 7/9, 199034 St Petersburg, Russia;
[3] Biological Department, Saint Petersburg State University, Universitetskaya Emb. 7/9, 199034 St Petersburg, Russia;
[4] Biological Department, Lomonosov Moscow State University, Leninskiye Gory 1, 119991 Moscow, Russia
[5] International Biotechnological Center, Lomonosov Moscow State University, Leninskiye Gory 1, 119991 Moscow, Russia
[6] Department of Neurobiology and Anatomy, Wake Forest School of Medicine, Medical Center Boulevard, Winston-Salem, 27157 North Carolina, USA
* Corresponding author

Supplementary materials and methods

Guide cannulas for intracerebroventricular (ICV) injections were made as small as possible in order to avoid using screws for the fixation of cannulas with dental cement because mouse skull is thin (0,1-0,2 mm) making additional brain damage by screws unavoidable. As seen in Fig. S1, guide cannulas on the mouse head were small enough to prevent animals from knocking them off the head and any discomfort during movements.

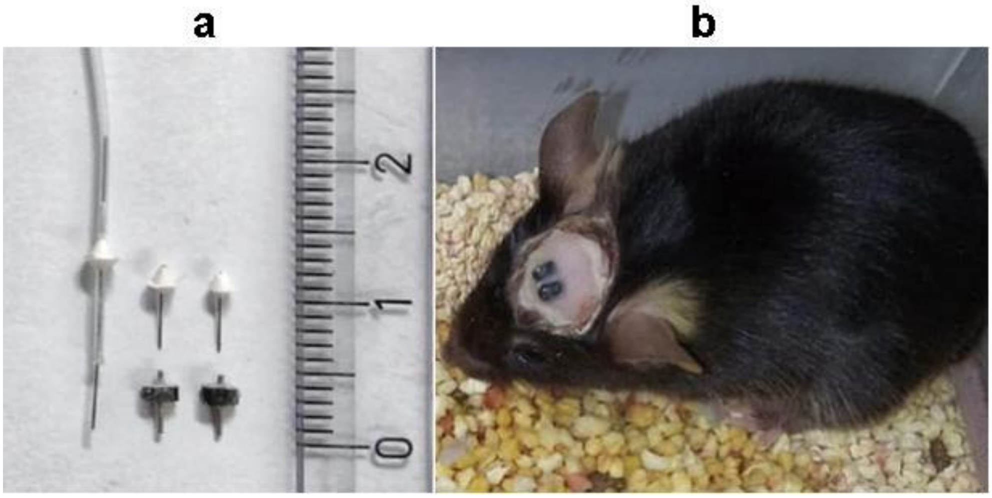

Supplementary Figure S1. Guide, dummy and injection cannulas design (a). Mouse with guide cannulas (after dummy cannulas withdrawing) (b).

Guide cannulas were placed at coordinates AP = –0.5, L = 1.0, depth 2.0 (coordinates are given in millimeters relative to the bregma [1]) to inject ouabain into the lateral ventricles directly above the striatum (Fig. S2).

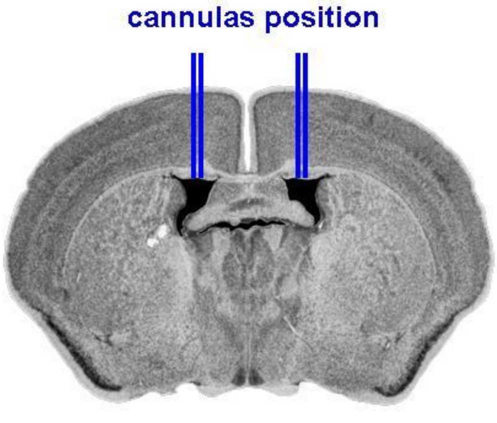

Supplementary Figure S2. Schematic image of guide cannulas position in the mouse brain (frontal slice [1]).

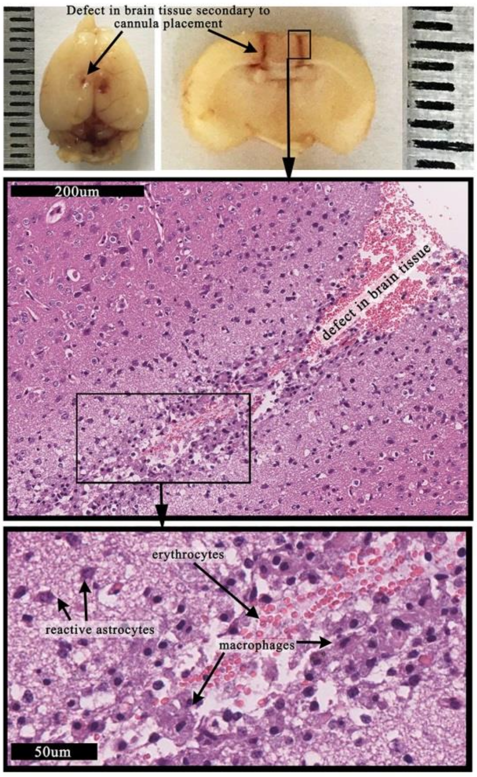

Supplementary Figure S3. Histological analysis of a frontal slice of an operated mouse on the level of ICV cannula implantation. The tissues were stained with hematoxylin and eosin.

It is visible that the guide cannula have reached the targeted lateral ventricles (Fig. S3). It is also visible on the histological microphotographs of the tissues that the guide cannula were placed in such a way that only the motor cortex in the area of the operation was damaged, and no significant damage was done to the nearby areas. No inflammation or edema was caused by the manipulations.

Supplementary results

Locomotor activity of mice after injection of different concentrations of ouabain

Preliminary experiments with different concentrations of ouabain were conducted to find the dose of ouabain that causes hyperactivity in mice. The groups of mice treated with other ouabain concentrations (10 μM and 200 μM) were small (N=3) to prevent unnecessary waste of animals. Fig. S4 is a modified Fig. 1 from the main text showing the effect of ouabain on the locomotor activity of mice with the addition of the results for other ouabain concentrations.

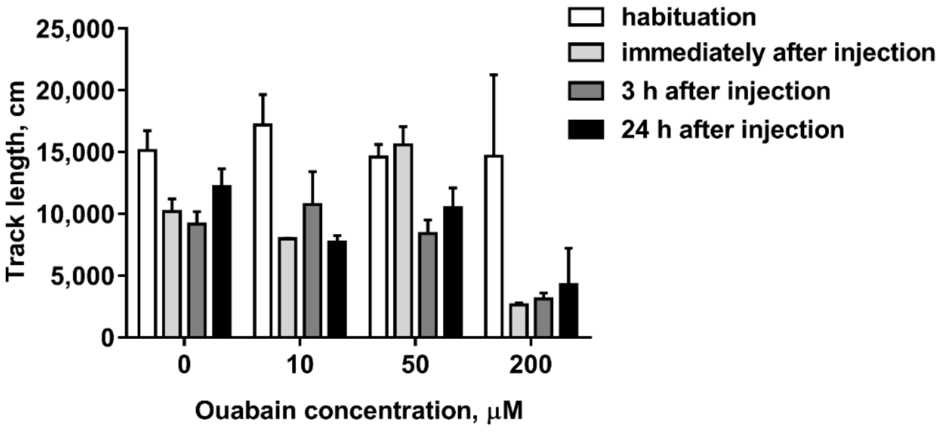

Supplementary Figure S4. Total distance travelled by mice in 1h open field tests. Data is presented as mean ± SEM, N=10 per group for control and 50 μM ouabain, N=3 for groups administered 10 μM and 200 μM ouabain.

Bilateral injection of 0,5 μl of 10 μM ouabain did not cause any effect on the locomotor activity of mice. Bilateral injection of 0,5 μl of 200 μM ouabain caused decreased locomotor activity and "freezing" of the animals (Fig. S4).

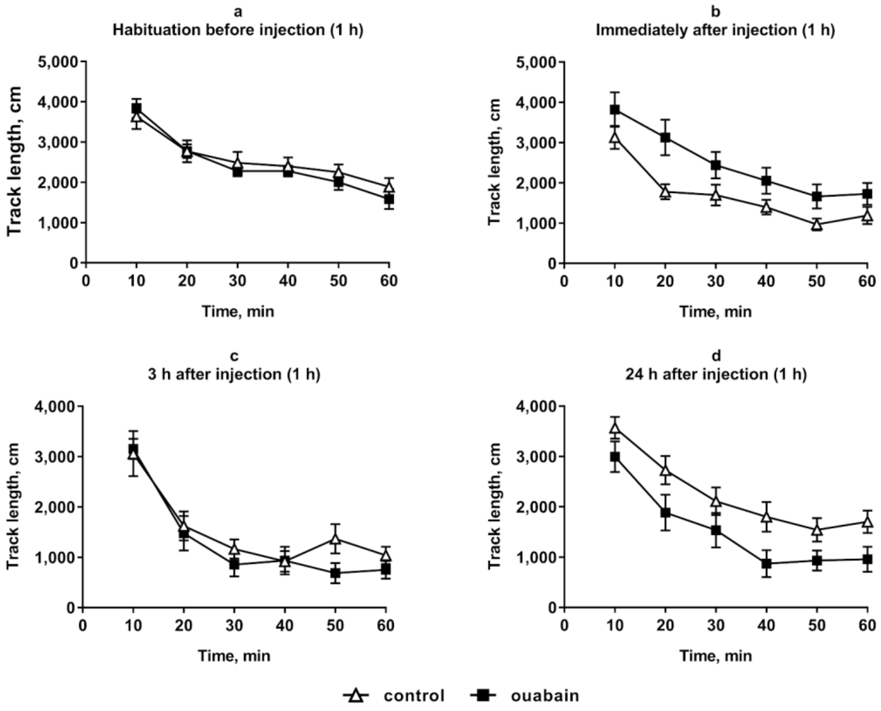

Supplementary Figure S5. Locomotor activity of mice in the open field test. Distance travelled by mice, calculated separately for every 10 min of a 1h open field test during habituation (a), immediately after the injection (b), 3 h after the injection (c), and 24 h after the injection (d). Data is presented as mean ± SEM, N=10 per group.

In all three open field tests, as well as during habituation, locomotor activity subsequently decreased with time both in the control and in ouabain-treated groups: time factor for habituation p<0,0001, F (5, 118) = 18,5 (Fig. 2a); time factor for 1 h after injection p<0,0001, F (1, 120) = 20,92 (Fig. 2b); time factor for 3 h after injection p<0,0001, F (5, 96) = 21,21 (Fig. 2c); time factor for 24 h after injection p<0,0001, F (1, 96) = 20,22 (Fig. 2d). There was no difference in locomotor activity between groups during habituation, group factor p=0,3892, F (1, 118) = 0,7468 (Fig. 2a) and during 1 h open field test 3 h after injection, group factor p=0,1549, F (1, 96) = 2,056 (Fig. 2c). The difference between ouabain-treated and control groups was observed throughout the 1h test immediately after injection, group factor p<0,0001, F (1, 120) = 20,92 (Fig. 2b) and also after 24 h, p<0,0001, F (1, 96) = 20,22 (Fig. 2d). The comparisons were analyzed using two-way ANOVA with post hoc Tukey.

During the 20 minutes post administration in the control group 33 ± 6 rearings were observed, while in the ouabain-treated group 65 ± 15 rearings were observed.

1h track visualization of a control mouse and 50 μM ouabain-treated mouse illustrates stereotypical movements of ouabain-treated mice in the open field test (Fig. S6).

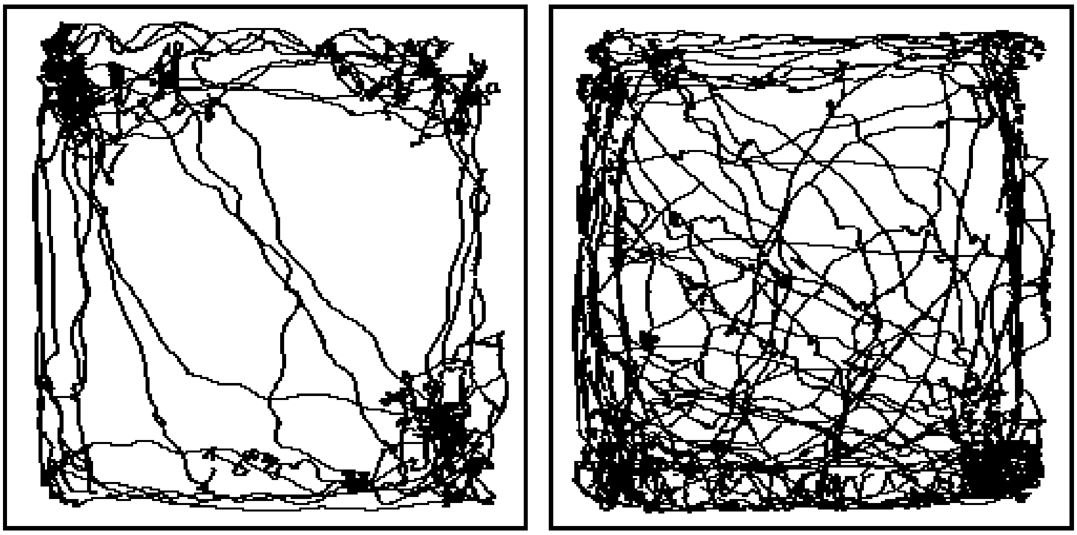

Supplementary Figure S6. 20-min track visualization of a control mouse (left) and 50 μM ouabain-treated mouse (right).

Catecholamine content in the striatum after ICV ouabain administration

As seen in Fig. S7, ouabain administration caused 3,4-dihydroxyphenylacetic acid and homovanillic acid levels in the striatum of mice to increase without any effect on the level of dopamine.

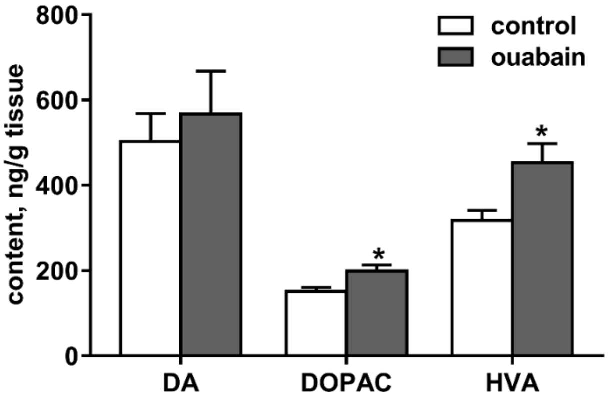

Supplementary Figure S7. The effect ouabain (ICV, 0,5 μl, 50 μM) on the content of dopamine (DA), 3,4-dihydroxyphenylacetic acid (DOPAC) and homovanillic acid (HVA) in the striatum of mice. Results are presented as mean ± SEM, N=8 per group; * – $p<0.05$ compared to control.

Akt, GSK3β and ERK1/2 activation in the striatal tissue of the experimental animals

To determine the influence of D2 dopamine receptor activation on ouabain-induced intracellular signal cascades in the striatum of mice, Akt, GSK3β and ERK1/2 activation was evaluated 30 min after the bilateral ICV injection of 0.5 μl of 50 μM ouabain and 0.5 μl of 200 μM ouabain (i. e. 1 h after intraperitoneal administration of haloperidol, 70 μg/kg).

Since the 200 μM ouabain group was not included in the data set in the main article, the full set of representative bands is presented in the supplementary materials. As you can see on

Supplementary Figure S8, 200 μM ouabain has the same influence on the activation of Akt, GSK3β and ERK1/2 as 50 μM ouabain.

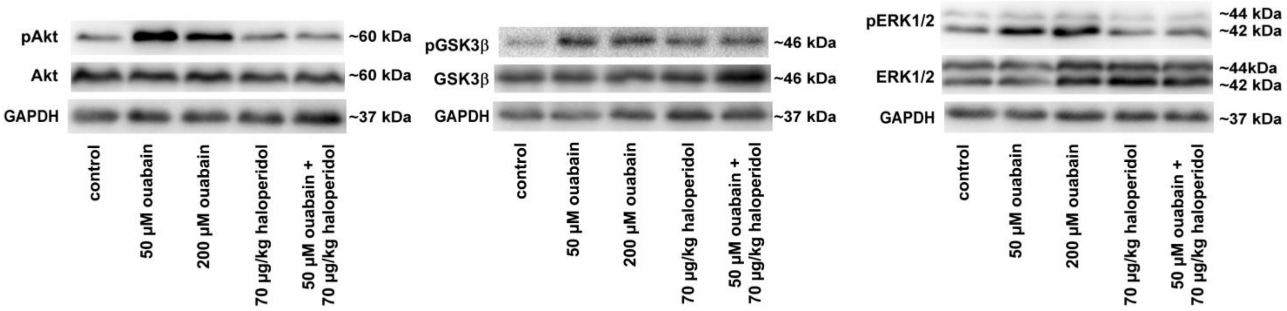

Supplementary Figure S8. Akt, GSK3β and ERK1/2 activation in the striatum of mice under combined or separate administration of ouabain and haloperidol. Representative bands of Akt, GSK3β and ERK1/2 phosphorylation in the striatal tissue of mice.

Supplementary references

1. Lein, E. S. *et al.* Genome-wide atlas of gene expression in the adult mouse brain. *Nature* **445**, 168-176, doi:10.1038/nature05453 (2007).
